# Histopathologic degenerative score as a predictor of minimal clinically important difference in pain and functionality following surgical treatment for disc herniation

**DOI:** 10.17305/bb.2024.10877

**Published:** 2024-07-20

**Authors:** Hakija Bečulić, Emir Begagić, Sabina Šegalo, Fatima Juković-Bihorac, Emsel Papić, Ragib Pugonja, Amina Džidić-Krivić, Adem Nuhović, Goran Lakičević, Semir Vranić, Mirza Pojskić

**Affiliations:** 1 Department of Neurosurgery, Cantonal Hospital Zenica, Zenica, Bosnia and Herzegovina; 2 Department of Anatomy, School of Medicine, University of Zenica, Zenica, Bosnia and Herzegovina; 3 Department of General Medicine, School of Medicine, University of Zenica, Zenica, Bosnia and Herzegovina; 4 Department of Laboratory Technologies, Faculty of Health Studies, University of Sarajevo, Sarajevo, Bosnia and Herzegovina; 5 Department of Pathology, Cantonal Hospital Zenica, Zenica, Bosnia and Herzegovina; 6 Department of Neurology, Cantonal Hospital Zenica, Zenica, Bosnia and Herzegovina; 7 Department of General Medicine, School of Medicine, University of Sarajevo, Sarajevo, Bosnia and Herzegovina; 8 Department of Neurosurgery, University Hospital Mostar, Zenica, Bosnia and Herzegovina; 9 College of Medicine, QU Health, Qatar University, Doha, Qatar; 10 Department of Neurosurgery, University Hospital Marburg, Marburg, Germany

**Keywords:** Degenerative disc disease, histopathology, intervertebral disc displacement, pain measurement, treatment outcome

## Abstract

Lumbar disc herniation (LDH) often results in significant pain and disability, and histopathologic (HP) evaluation of intervertebral discs (IVDs) offers critical insights into treatment outcomes. This prospective observational study explores HP changes in IVDs and their association with clinical outcomes following surgical treatment for LDH. A cohort of 141 patients undergoing MRI-confirmed LDH surgery underwent HP evaluation using a semi-quantitative HP degeneration score (HDS). Preoperatively and at a six-month follow-up, the comprehensive clinical assessment included the Oswestry disability index (ODI) and visual analog scale (VAS), with a minimal clinically important difference (MCID) calculated from ODI and VAS. Results indicated significant associations between higher HDS and adverse clinical outcomes, including persistent pain and greater disability post-surgery. Specifically, an HDS ≥ 7 was predictive (OR ═ 6.25, 95% CI: 2.56–15.23) of disability outcomes measured with MCID-ODI (AUC: 0.692, 95% CI: 0.609–0.767, *P* < 0.001), and HDS ≥ 8 was predictive (OR ═ 1.72, 95% CI: 1.04–2.77) of persistent pain measured with MCID-VAS (AUC ═ 0.628, 95% CI: 0.598–0.737, *P* ═ 0.008), highlighting the diagnostic potential of HDS in assessing postoperative recovery. This study underscores the potential of HP evaluation using HDS to provide valuable insights into disease progression and outcomes in LDH patients, complementing conventional radiologic methods. The findings support the application of personalized treatment strategies based on HP findings while acknowledging challenges in interpretation and clinical implementation.

## Introduction

The intervertebral disc (IVD) is a specific and vulnerable anatomical structure that is subject to degenerative processes and changes caused by various factors such as load, range of motion of the lumbar spine, and localization [1]. IVD degeneration (IDD) is the main cause of lower back pain, and one of the main causes of disability [2, 3]. The basis for the occurrence of degenerative disc disease is formed from birth and consists of a series of complex pathophysiologic processes that contribute to the development of degenerative spinal diseases such as lumbar disc herniation (LDH) [4, 5].

Specific changes in the biology of the IVD have been associated with LDH. These changes include decreased water retention in the *nucleus pulposus* (NP), increased content of type I collagen in the NP and inner *annulus fibrosus* (AF), degradation of extracellular matrix components, activation of apoptosis, expression of matrix metalloproteinases, and activation of inflammatory pathways [6–8]. Of particular note is the link between inflammatory signaling and nerve pain in LDH, which underscores the immunologic privileging of IVD [9, 10]. As the NP protrudes into the epidural space, changes in vascular permeability and vasodilation promote the recruitment of immune cells and the release of inflammatory cytokines [11, 12]. Histological characteristics associated with IDD include increased cell density (chondrocyte proliferation), frequent granular changes, structural alterations (tears and fissures), and a significant accumulation of mucopolysaccharides (mucinous degeneration) with dark blue areas surrounding chondrocyte clusters [13]. These parameters formed the basis for the development of a semiquantitative HP degenerative score (HDS) by Boos et al. [14]. Despite being explored in a limited number of studies, histologic analysis of IVD offers valuable insights into the pathophysiological mechanisms and histopathologic (HP) changes that are often not detectable by conventional radiologic methods [15].

This study investigates how HP changes in IVDs correlate with clinical findings and outcomes following surgical treatment for LDH. It explores the relationship between microscopic alterations in disc tissues and post-surgical pain management and functional recovery. By analyzing these HP findings, the study evaluates their prognostic significance and potential to guide personalized treatment approaches for LDH patients undergoing surgery. The research aims to enhance prognostic tools beyond conventional diagnostic methods like magnetic resonance imaging (MRI), providing deeper insights into the underlying mechanisms of LDH to optimize patient outcomes.

## Materials and methods

### Study design, sample size, and subjects

This prospective observational study included patients who had undergone surgery for a hernia of the IVD at the Department of Neurosurgery of the Cantonal Hospital Zenica (Bosnia and Herzegovina) between July 2022 and June 2023. Patients were included if they required surgery due to LDH, were older than 18 years, were residents of the Zenica-Doboj Canton (ZDC), and had MRI records and data available. Patients were excluded if they had lumbar spine trauma, spondylolisthesis, recurrent IVD prolapse, failed back surgery syndrome, or an infection.

The sample size was calculated using the prevalence of patients who underwent surgical treatment during 2022. The total number of patients with indications for surgical intervention was 143 in the ZDC, Bosnia and Herzegovina. According to the Federal Institute of Statistics in 2022, the total population in the ZDC was 364,433 (https://fzs.ba/). The sample size was determined using the following equation [16]:
(1)
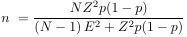

where *n* represents the sample size, *N* is the total population, *Z* is the value for a 95% confidence level (*Z* ═ 1.96), *p* is the estimated prevalence rate in the population (*p* ≈ 0.000395), and *E* represents the desired precision (*E* ═ 0.00005). Based on the calculation, the representative sample size is 141 patients.

### Data collection

The dataset from medical records included demographic, occupational, lifestyle, and clinical variables. Gender was categorized as male or female. Age was recorded both continuously and categorically, with groups formed using the median age. Education was categorized into elementary, secondary, and college, and living environments into urban and rural. Vertebral bodies (L2/L3, L3/L4, L4/L5, L5/S1) were examined via MRI (Magnetom Avanto 1.5 T, Siemens, Erlangen, Germany). Anthropometric measures included height (meters), weight (kilograms), and body mass index (BMI) in kg/m^2^. BMI categories were defined as underweight (<18.5), normal weight (18.5–24.9), overweight (25–29.9), and obese (≥30) [17]. Before surgery, workplace data and occupational risks were gathered from anamnestic records, including details on shift work, overtime, employment sector, income, employment duration, tobacco and alcohol use, physical activity, and sleep duration. Comorbidities included type 1 or type 2 diabetes, hypertension, depression, disc herniation at another level, lumbar spinal stenosis, or autoimmune conditions, confirmed by a specialist physician.

### HP analysis

**Figure 1.**
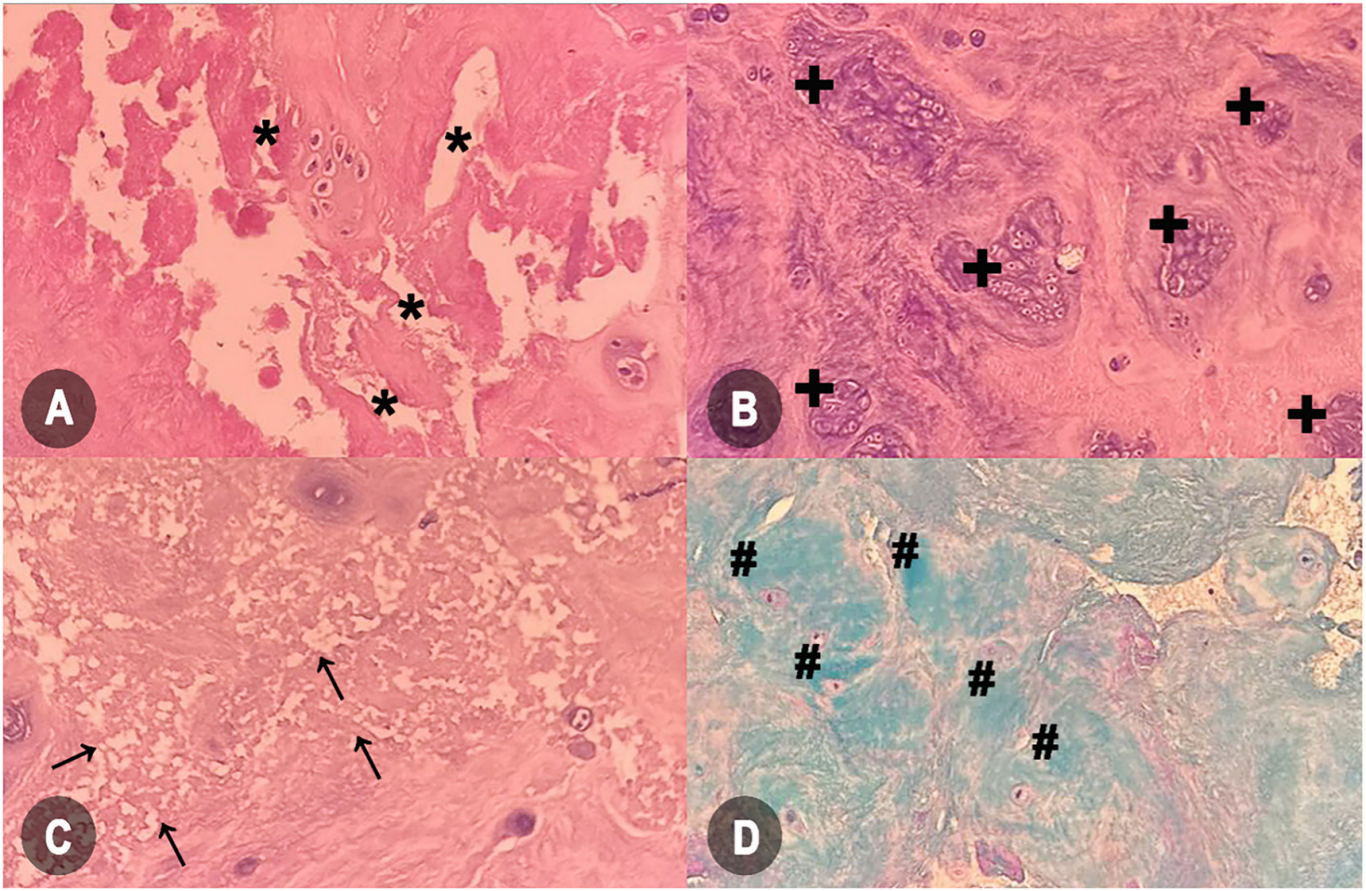
**Microscopic features illustrating intervertebral disc degeneration.** (A) Structural modifications marked by tears and clefts (marked with *); (B) Increase in cell density, signifying chondrocyte proliferation granular alterations (marked with +); (C) Granular alterations (marked with arrows); (D) Significant escalation in acid mucopolysaccharides, indicating mucous degeneration (marked with #) (Images A, B, and C are hematoxylin and eosin stained; image D is Alcian blue stain; magnification 20×).

A posterior lumbar discectomy was performed to remove extruded or sequestered disc material (DM) from the patient. The excised DM was preserved and sent for HP examination. For analysis, the specimen was placed in a 4% formaldehyde solution with a pH of 7.4 for 12–16 h. Thin sections (4 µm) were prepared from the formalin-fixed material, embedded in paraffin, placed on adequate glass slides, and stained with hematoxylin and eosin (H&E) and Alcian blue to determine the changes in tissue morphology using a semiquantitative method, the HDS [14]. For each subscale, values were assigned as follows (Figure 1): cell density (chondrocyte proliferation) with scores ranging from 0 (no evidence of proliferation) to 5 (presence of significant clusters >15 cells); structural changes (tears and clefts) with scores ranging from 0 (absence) to 4 (evidence of scarring or tissue defects); granular changes and mucosal degeneration with scores ranging from 0 (absence) to 3 (marked presence). The evaluation provided information on the degree of degeneration on an HDS scale of 0–15, calculated by summing the values of the subscales.

### Clinical evaluation and follow-up

The clinical evaluation included preoperative and follow-up assessments of pain intensity, motor and sensory function, and disability due to disc herniation. Follow-up was conducted six months (±15 days) post-surgery. Pain was measured with the visual analog scale (VAS) from 0 to 10, where higher scores indicate greater pain intensity [18]. Motor function (MF) was assessed using the Medical Research Council Muscle Power Scale (MRC-MPS) with the following ratings: 0 – no visible contraction, 1 – visible minimal contraction, 2 – movement without overcoming gravity, 3 – active movement overcoming gravity, 4 – movement overcoming some resistance, and 5 – normal strength [19]. Sensory impairment was assessed using a sensitivity assessment scale (SAS) for L1-S3 dermatomes with values ranging from 0 (absent) to 2 (normal) [20]. Disability caused by disc herniation was evaluated using the Oswestry disability index (ODI) scale, consisting of ten domains rated on a Likert-type scale from 0 to 5. The total score ranges from 0 to 50, with a higher score indicating greater disability among the patients under investigation [21]. Pain levels were gauged using VAS, ranging from 0 to 10, where higher scores denoted more intense pain. The minimal clinically important difference (MCID) system, established by Power et al. [22], was employed to assess diagnostic precision. This system identified a decrease of 22 points in ODI scores post-surgery compared to preoperative levels as indicative of a positive outcome. MCID was similarly computed for VAS values, with a reduction of 2.5 points signifying a beneficial result [23].

### Ethical statement

Ethical approval was obtained from the Ethics Committee of the Zenica Cantonal Hospital (number 00-03-35-915-8/22). All patients were informed about the purpose and significance of the study and signed a written informed consent form. The personal data of the patients were protected, and the principles of the Declaration of Helsinki were followed.

### Statistical analysis

The Statistical Package for the Social Sciences (SPSS) software (IBM Inc., USA, version 27.0) and MedCalc (MedCalc Software, Ostend, Belgium, version 22) were used for the statistical analysis. Deviations from a normal distribution were assessed using the Kolmogorov–Smirnov test. Significant differences in categorical variables were determined using the chi-square test, while the Wilcoxon test was used for continuous variables. The influence of the above variables on the degree of degeneration measured by HDS was assessed by multivariate or logistic regression analysis with odds ratio (OR) with a 95% confidence interval (CI). For continuous variables, linear regression was performed. Diagnostic accuracy was evaluated using receiver operating characteristic (ROC) curve analysis, specifically through the calculation of the area under the curve (AUC). Statistical significance was set at *P* ≤ 0.05.

## Results

The dataset included 83 men (58.9%) and 58 women (41.1%) with a median age of 44 years (IQR: 37–55). A majority of patients (52.5%) were older than 44 years. Educationally, 18.4% had completed primary school, 53.9% secondary school, and 27.7% college. Geographically, 44.0% were from urban areas and 56.0% from rural areas. The median height was 1.8 m (IQR: 1.7–1.8), weight was 80.5 kg (IQR: 66.6–89.8), and BMI was 25.0 kg/m^2^ (IQR: 23–28). Among them, 50.1% were of normal weight, 40.4% were overweight, and 9.2% were obese. Disc herniation occurred in 22.0% at L3/L4, 46.8% at L4/L5, and 31.2% at L5/S1.

**Figure 2.**
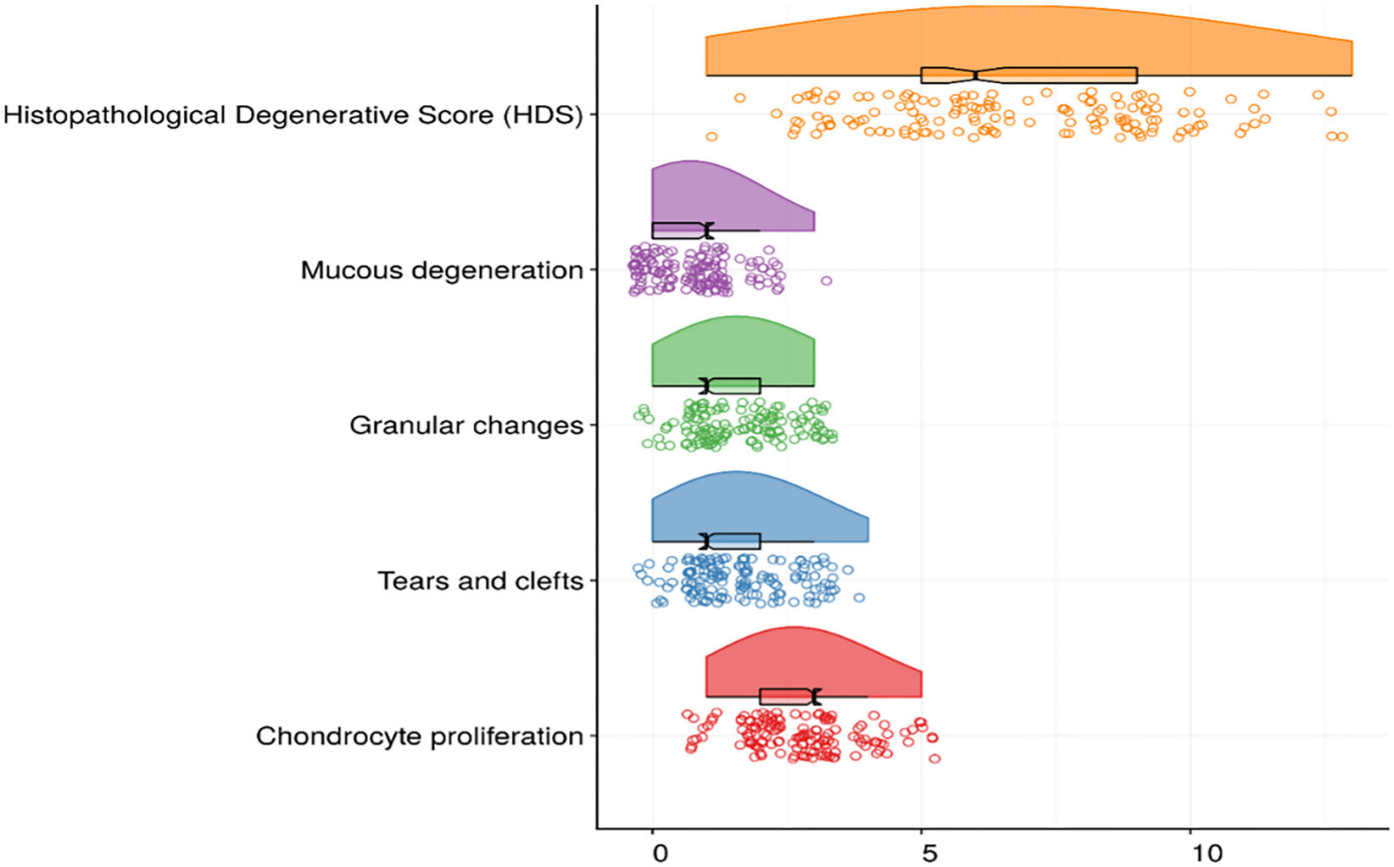
Results of histopathologic analysis of intervertebral discs with median values of domains and total histopathological degenerative score.

HP analysis indicated chondrocyte proliferation (median: 3, IQR: 2–3), tissue tears and clefts (median: 1, IQR: 1–2), granular changes, and mucous degeneration (median: 1, IQR: 0–1), and an HDS median of 6 (IQR: 5–9) (Figure 2). Males (OR ═ 2.21, 95% CI: 1.78–2.99, *P* < 0.001) and patients under 44 years (OR ═ 1.97, 95% CI: 1.59–2.45, *P* < 0.001) had higher odds of HDS. Greater height (β ═ 0.01, 95% CI: 0.00–0.01, *P* ═ 0.002), weight (β ═ 0.11, 95% CI: 0.09–0.14, *P* < 0.001), and BMI (β ═ 0.98, 95% CI: 0.83–1.14, *P* < 0.001) were linked to higher HDS scores. Overweight (OR ═ 2.19, 95% CI: 1.69–2.83, *P* < 0.001) and obesity (OR ═ 2.24, 95% CI: 1.50–3.34, *P* < 0.001) were also associated with increased odds of HDS. LDH at L4/L5 (OR ═ 2.54, 95% CI: 1.69–3.82, *P* < 0.001) and L5/S1 (OR ═ 1.55, 95% CI: 1.27–1.89, *P* < 0.001) showed significant associations with HDS (Table 1).

**Table 1:** Baseline characteristics of the cohort

			**Regression analysis (HDS)**	
**Variable**		***N* (%) or Median (Q1:Q3)**	**OR or β (95% CI)**	***P* value**
Gender	Men	83 (58.9)	2.21 (1.78; 2.99)	<0.001
	Women	58 (41.1)	Reference	–
Age (years)		47.0 (37.0–55.0)	0.15 (0.12; 0.18)*	<0.001
Age (categories)	≥44	74 (52.5)	Reference	–
	<44	67 (47.5)	1.97 (1.59; 2.45)	<0.001
Education	Primary school	26 (18.4)	1.53 (0.76; 1.90)	0.249
	Secondary school	76 (53.9)	1.23 (0.51; 1.77)	0.457
	College/Faculty	39 (27.7)	Reference	–
Life environment	Urban	62 (44.0)	1.43 (0.84; 1.64)	0.365
	Rural	79 (56.0)	Reference	–
Height (m)		1.8 (1.7–1.8)	0.01 (0.00; 0.01)*	0.002
Weight (kg)		80.5 (66.6–89.8)	0.11 (0.09; 0.14)*	<0.001
BMI (kg/m^2^)		25.0 (23.0–28.0)	0.98 (0.83; 1.14)*	<0.001
BMI (categories)	Normal weight	71 (50.1)	Reference	–
	Overweight	57 (40.4)	2.19 (1.69; 2.83)	<0.001
	Obesity	13 (9.2)	2.24 (1.50; 3.34)	<0.001
Affected vertebral level	L3/L4	31 (22.0)	Reference	–
	L4/L5	66 (46.8)	2.54 (1.69; 3.82)	<0.001
	L5/S1	44 (31.2)	1.55 (1.27; 1.89)	<0.001

*: β coefficients along with 95% CIs (derived from linear regression analysis), while the remaining values not marked with “*” represent OR with 95% CIs obtained through logistic regression analysis or multinomial regression analysis. HDS: Histopathological degenerative score; BMI: Body mass index.

**Figure 3.**
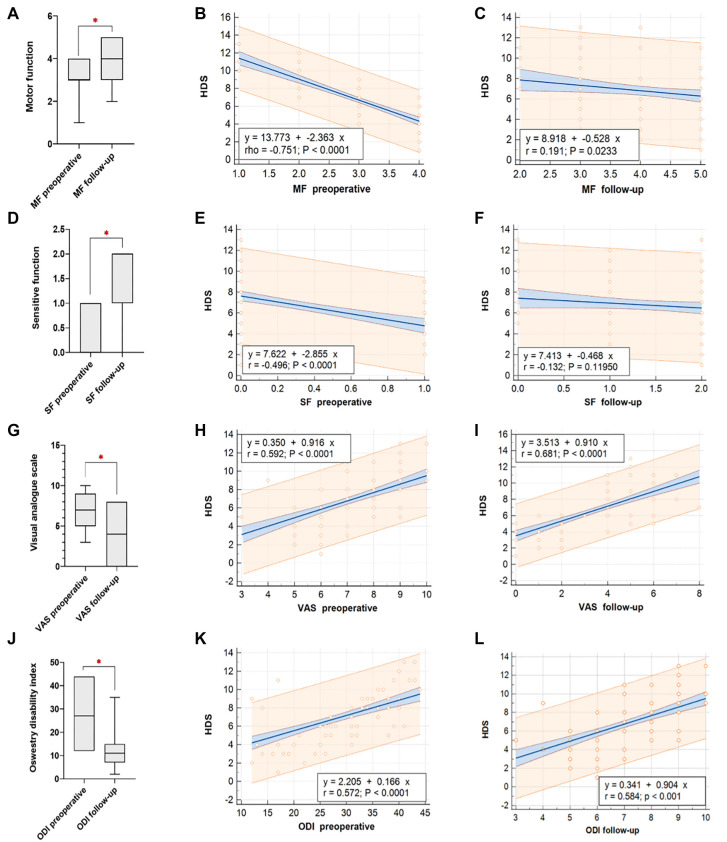
**(A) Comparative analysis of MF, (D) SF, (G) VAS, (J) ODI both before surgery and during subsequent follow-up evaluations.** Correlations between the Health-related Quality of Life, Determination score (HDS) and preoperative MF are depicted in (B), while (C) depicts correlations during the follow-up MF assessment. The correlation between SF preoperatively and at follow-up is displayed in (E) and (F). (H) and (I) represent the correlation of VAS scores with preoperative and follow-up assessments respectively. Lastly, (K) and (L) present the correlation between ODI scores and HDS both before surgery and during follow-up evaluations. MF: Motor function; SF: Sensitive function; VAS: Visual analogue scale; ODI: Oswestry disability index; HDS: HP degeneration score.

The median MF scores increased from 3 (IQR: 3–4) to 4 (IQR: 3–5) (*P* ═ 0.001), and sensitive function (SF) scores from 1 (IQR: 1–2) to 2 (IQR: 1–2) (*P* ═ 0.003). MF scores showed a strong negative correlation with HDS preoperatively (*r* ═ −0.751, *P* < 0.001) (Figure 3B), while SF scores demonstrated a moderate negative correlation (*r* ═ −0.469, *P* < 0.001) (Figure 3E). Both MF and SF correlations with HDS decreased in follow-up examinations (Figure 3C and 3F). VAS decreased from a median of 7 (IQR: 5–9) preoperatively to 4 (IQR: 2–5) postoperatively (*P* < 0.001) (Figure 3G). ODI decreased from a median of 27 (IQR: 19–35) to 11 (IQR: 7–15) (*P* < 0.001) (Figure 3J). Higher preoperative VAS scores were associated with increased follow-up HDS (*r* ═ 0.592, *P* < 0.001), as were higher ODI scores (*r* ═ 0.584, *P* < 0.001) (Figure 3L).

Work in shifts (*B* ═ 0.757, *P* ═ 0.012), longer employment durations (*B* ═ 1.203, *P* < 0.001), tobacco consumption (*B* ═ 2.911, *P* < 0.001), and physical activity (B ═ −0.919, *P* ═ 0.005) were associated with HDS (Table 2). Factors such as diabetes mellitus (*B* ═ 1.341, *P* ═ 0.028), hypertension (*B* ═ 1.502, *P* < 0.001), depression (*B* ═ 1.590, *P* ═ 0.038), and spinal lumbar stenosis (*B* ═ 2.349, *P* < 0.001) were associated with higher HDS scores (Table 3).

**Table 2:** Factors related to occupation, behavior, and habits

			**Multivariate regression analysis (HDS)**	
**Variable**		***N* (%)**	**β coefficient (95% CI)**	***P* value**
Work in shifts	No	44 (31.2)	0.757 (0.166; 1.348)	0.012
	Yes	97 (68.8)		
Overtime work	No	66 (46.8)	0.504 (−0.047; 1.060)	0.073
	Yes	75 (53.2)		
Sector	Public	78 (55.3)	0.521 (−0.018; 1.060)	0.058
	Private or self-employed	63 (44.7)		
Income	Below average	75 (53.2)	−0.472 (−1.030; 0.867)	0.097
	Above average	66 (46.8)		
Length of employment (years)	<5	26 (18.4)	1.203 (0.752; 1.654)	<0.001
	6–15	76 (53.9)		
	>16	39 (27.7)		
Tobacco consumption	No	70 (49.6)	2.911 (2.195; 3.626)	<0.001
	Yes	71 (50.4)		
Alcohol consumption	No	55 (39.0)	0.107 (−0.426; 0.639)	0.693
	Yes	86 (61.0)		
Physical activity	No	102 (72.3)	−0.929 (−1.572; −0.286)	0.005
	Yes	39 (27.7)		
Sleep (hours)	<7 hours	59 (41.8)	−0.171 (−0.820; 0.478)	0.603
	≥7 hours	82 (58.2)		

HDS: Histopathologic degenerative score; CI: Confidence interval.

**Table 3:** Factors related to comorbidities

			**Multivariate regression analysis (HDS)**	
**Variable**		***N* (%)**	**β coefficient (95% CI)**	***P* value**
Diabetes mellitus	No	110 (78.0)	1.341 (0.149; 2.533)	0.028
	Yes	31 (22.0)		
Hypertension	No	80 (56.8)	1.502 (0.820; 2.184)	<0.001
	Yes	61 (43.3)		
Depression	No	129 (91.5)	1.590 (0.092; 3.089)	0.038
	Yes	12 (8.5)		
Disc herniation on other levels	No	78 (55.3)	0.570 (−0.184; 1.324)	0.137
	Yes	63 (44.7)		
Spinal lumbar stenosis	No	124 (87.9)	2.349 (1.124; 3.574)	<0.001
	Yes	17 (12.1)		
Other autoimmune diseases	No	133 (94.3)	1.489 (−0.072; 3.050)	0.061
	Yes	8 (5.7)		

HDS: Histopathologic degenerative score; CI: Confidence interval.

**Figure 4.**
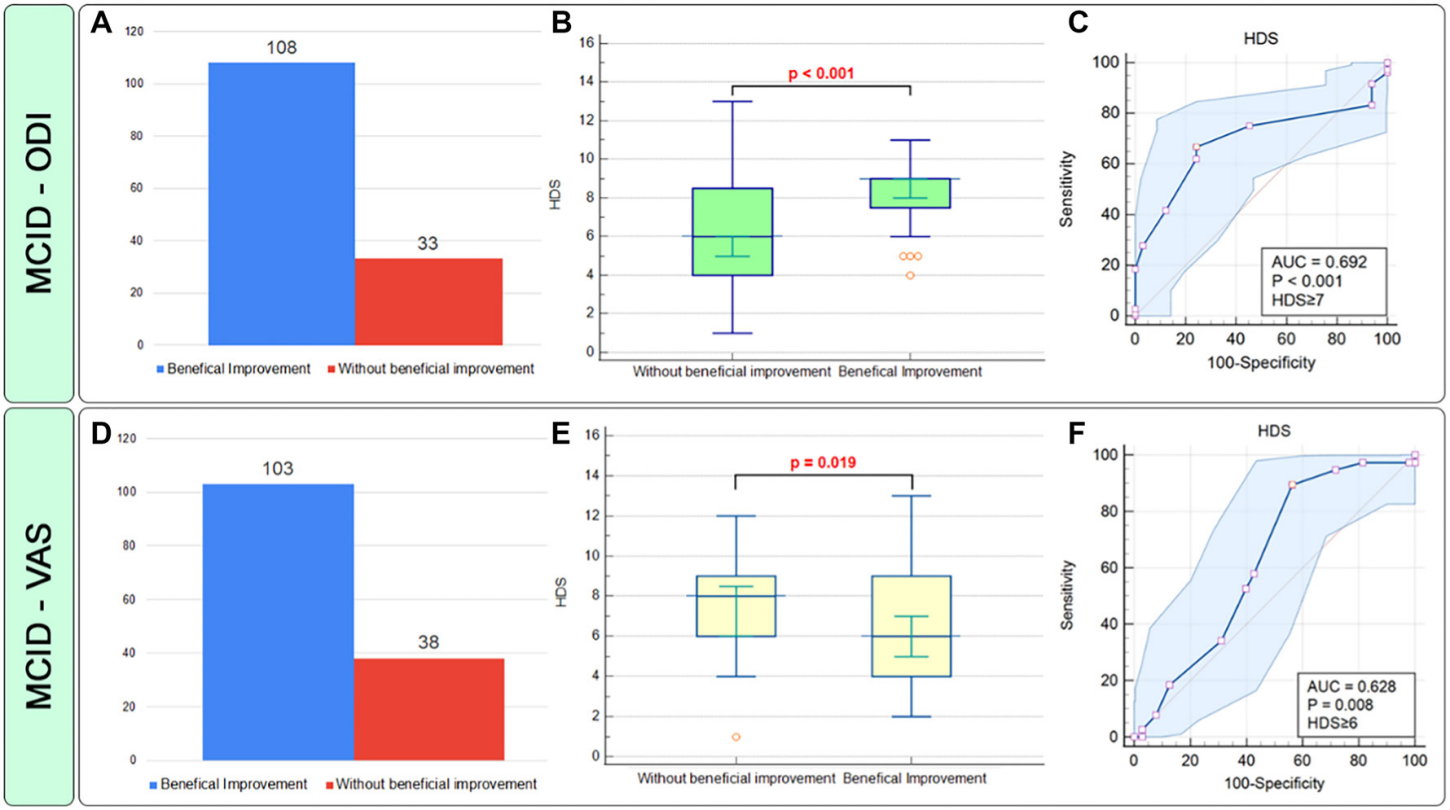
**Prognostic accuracy evaluation of the histopathological degeneration score (HDS) utilizing the MCID criteria, assessed through the ODI and visual analog scale (VAS) over a six-month follow-up period.** The figure depicts: (A) Frequencies of beneficial improvement concerning MCID–ODI; (B) HDS values based on MCID (ODI) and Receiver Operating Characteristic (ROC) curve analysis; (C) Frequencies of beneficial improvement concerning MCID–VAS; (D) HDS values categorized by MCID–VAS groups, and (E) ROC analysis based on MCID–VAS. HDS: Histopathological degeneration score; MCID: Minimal clinically important difference; VAS: Visual analogue scale; ODI: Oswestry disability index; ROC: Receiver operating characteristic.

In the ODI-measured MCID, 76.6% of patients showed improvement at six months. Participants who improved had a median HDS score of 9.0 (95% CI: 9.0–11.0), while those who did not had a median score of 6.0 (95% CI: 6.0–8.0) (Figure 4B). The AUC was 0.692 (95% CI: 0.609–0.767), significant at *P* < 0.001 (Figure 4C). An HDS criterion of ≥ 7 had a sensitivity of 89.47% and a specificity of 43.69%. A significant association was found between HDS ≥ 6 and MCID-ODI (OR ═ 6.25, 95% CI: 2.56–15.23). In VAS-assessed MCID, 73% of patients showed improvement. Those without improvement had a median HDS of 8.0 (95% CI: 8.0–9.0), compared to 6.0 (95% CI: 6.0–8.0) for those with improvement (*P* ═ 0.019) (Figure 4E). The AUC was 0.628 (95% CI: 0.598–0.737, *P* ═ 0.008) (Figure 4F), with an HDS criterion of ≥ 6, having 66.67% sensitivity and 75.76% specificity. A significant association was found between HDS ≥ 6 and MCID-VAS (OR ═ 1.72, 95% CI: 1.04–2.77).

## Discussion

In our study, the HDS emerged as a potential predictor of patient outcomes following surgical intervention for LDH. We observed that an HDS threshold of ≥ 7 exhibited good diagnostic and prognostic capabilities in evaluating patient disability outcomes. Additionally, HDS ≥ 8 was significantly associated with the persistence of pain six months post-treatment. To our knowledge, this is the first study to establish MCID-based cut-off values for HDS, specifically related to disability and pain outcomes.

The results of this study reveal a statistically significant correlation between HDS and motor and sensory function, as well as with the degree of pain and disability in patients undergoing surgical treatment for disc herniation. These findings align with previous research highlighting the intricate relationship between HP findings and clinical outcomes. For example, Oprea et al. [24] demonstrated that the mean surface area of chondrons, a marker of disc degeneration, correlated with lumbar VAS scores (*r* ═ 0.376) and moderately with Modic Changes (*r* ═ 0.500), findings that closely correspond with the results observed here. Moreover, Oprea et al. [24] identified a significant association between the degree of IVD degeneration and the Japanese Orthopedic Association Score (JOAS), which assesses neuromuscular function, supporting the predictive value of HDS. Similarly, Middendorp et al. [25] found a correlation between ODI scores and the grade of disc degeneration, further substantiating the impact of HDS on patient disability outcomes. Peletti-Figueiró et al. [26] and Bečulić et al. [27] independently confirmed a positive correlation between the extent and progression of IVD degeneration, respectively, reinforcing the notion that disease progression significantly influences LDH outcomes. However, not all studies agree with these findings; for instance, Willburger et al. [28] reported no significant correlation between sensory impairment and motor strength with the histologic composition of herniated material. Addressing the inflammatory etiology of pain is crucial in the conservative postoperative treatment of disc herniation patients, as emphasized by Lyu et al. [29]. Additionally, experimental studies such as those by Willburger et al. [28] and Ishikawa et al. [30] provide further evidence of the link between degenerative changes in DM and the severity of pain experienced by patients, supporting the clinical relevance of these findings.

To the best of our knowledge, this is the first study to identify factors related to the severity of disc degeneration based on HP analysis. This research revealed that HDS is associated with various risk factors, including sex, age, anthropometric measures, and occupational exposures, providing a novel perspective on the interplay between microscopic degenerative changes in DH. These risk factors have previously been linked to the severity of symptoms caused by LDH, predominantly through radiological findings [31–34]. This comparison with HP analysis and HDS scoring supports this method as a valuable approach for evaluating the severity of IVD degeneration.

HP analysis emerges as a valuable adjunctive tool in understanding the complexities of LDH, particularly in cases where surgical intervention may not be feasible. By discerning correlations between histologic findings and patient demographics, including age, gender, and anthropometric measures, this approach offers tailored insights into LDH pathogenesis, especially in inoperable or limited surgical option cases [35, 36]. Furthermore, HP analysis enables a personalized medicine approach by correlating histologic observations with clinical parameters such as motor and sensory function, pain intensity, and functional disability, thereby informing postoperative or post-biopsy pharmacological treatment strategies [37] to optimize patient care and outcomes.

Despite its utility, HP analysis presents inherent challenges. The invasive nature of tissue sampling procedures poses potential risks and discomfort to patients, necessitating careful consideration of ethical and safety concerns. Furthermore, the accessibility of HP facilities may be limited in certain healthcare settings, posing barriers to widespread implementation [38]. Additionally, the semiquantitative nature of histological findings introduces variability, necessitating standardized protocols and rigorous quality assurance measures. Moreover, the resource-intensive nature of HP analysis, both in terms of time and cost [38], may constrain its widespread application and necessitate judicious patient selection.

The study’s strengths include its prospective design, detailed inclusion criteria, and robust ethical considerations. However, limitations include the study’s single-center nature, which may limit generalizability, and the semi-quantitative method of HP analysis, which could introduce variability in results.

## Conclusion

In conclusion, our study establishes HDS as a potential predictor of post-surgical outcomes for LDH, with thresholds ≥ 7 and ≥ 8 indicating significant diagnostic and prognostic value for disability and pain persistence at six months. Despite its potential for personalized medicine insights, challenges such as procedural invasiveness, variability in HP interpretation, and resource constraints should be carefully considered.
